# Subacute Oral Toxicity of Yukmijiwhang-Tang in Crl:CD Sprague-Dawley Rats and Its Cytotoxicity 

**DOI:** 10.1155/2014/362573

**Published:** 2014-11-05

**Authors:** Soo-Jin Jeong, Chang-Seob Seo, Jung-Im Huh, Hyeun-Kyoo Shin

**Affiliations:** ^1^Herbal Medicine Formulation Research Group, Herbal Medicine Research Division, Korea Institute of Oriental Medicine, Daejeon 305-811, Republic of Korea; ^2^Division of Non-Clinical Studies, Korea Institute of Toxicology, Daejeon 305-343, Republic of Korea

## Abstract

*Background*. The traditional herbal formula Yukmijiwhang-tang (YMJ) consists of six medicinal herbs and has been used to treat dysuria, diabetic mellitus, and neurosis in Korea, China, and Japan. Here we report safety information on its subacute toxicity and the cytotoxicity. *Methods*. YMJ extract was administered to SD rats at various dosages for 4 weeks. We monitored clinical signs, mortality, body and organ weights, food intake, and hematological and serum biochemistry factors. For cytotoxicity testing, each cell line was treated with various concentrations of YMJ for 24 h. *Results*. YMJ treatment had no significant effects on changes in clinical signs, body weight, or food intake in male or female rats. In male rats, YMJ treatment decreased the absolute weights of the epididymides and serum Na levels. In female rats, YMJ significantly reduced the prothrombin time (PT) and serum creatine level. However, the changes were not severe and were considered to be in the normal physiological range for rats. The no-observed-adverse-effect-level (NOAEL) was estimated to be 2000 mg/kg/day. YMJ extract did not exert any cytotoxicity against 23 tested cell lines. *Conclusions*. Our data provide scientific evidence on the safety of YMJ for potential development as a prescription drug.

## 1. Introduction

Herbal medicine is a part of complementary and alternative medicine (CAM) including acupuncture, moxibustion, and other therapeutic methods. Traditionally, herbal medicine was used to restore a balance of energy in the body to maintain health rather than treat diseases. Many recent preclinical and clinical studies have provided scientific evidence on the efficacies of herbal medicines as therapeutic agents for various diseases. However, for drug development of herbal medicines, the safety information is scarce compared with synthetic chemical drugs. According to regulations of the United States Food and Drug Administration (FDA), dietary supplements including herbal products are different from Western prescription medicines or over-the-counter (OTC) drugs. Although herbal medicines are generally thought to be safe, some of them have toxicity and can induce drug interaction with others. Therefore, toxicity evaluation for herbal medicine should be considered as an important step prior to efficacy studies.

Yukmijiwhang-tang (YMJ; Rokumi-jio-gan in Japan, Liu Wei Di Huang tang in China) is a traditional herbal cocktail composed of six medicinal herbs: Rehmanniae Radix Preparata, Corni Fructus, Dioscoreae Rhizoma, Moutan Cortex, Poria Sclerotium, and Alismatis Rhizoma [1]. Basically, YMJ is a modified formula derived from Palmijihwang-tang (PMJ) by subtracting two herbs* Plantago asiatica* Linn. and* Cinnamomum cassia* Presl. YMJ was one of the most frequently prescribed herbal formulas found during the Song dynasty of China. YMJ has been used traditionally for the treatment of renal diseases, edema, diabetes, neurosis, and aging in Korea and China. Modern research has reported various biological activities of YMJ or YMJ derivatives (YMJD), such as antiosteoporotic [2, 3], antiamnesia [4, 5], antiasthmatic [6], and memory-enhancing [7] effects. In addition, our group recently reported the efficacy of YMJ treatment against benign prostatic hyperplasia* in vivo *[8].

Here we investigated the subacute toxicity of 4-week repeated oral doses of YMJ in a Crl:CD (SD) rat model according to guidelines established by the Organization for Economic Cooperation and Development (OECD) for the testing of chemicals in accordance with current Good Laboratory Practice regulations [9]. In addition, the cytotoxicity of YMJ was evaluated against various cell lines of different origins.

## 2. Materials and Methods

### 2.1. Chemicals and Reagents

5-(Hydroxymethyl)furfural (5-HMF) and gallic acid were purchased from Sigma-Aldrich (St. Louis, MO, USA). Loganin, paeoniflorin, and paeonol were obtained from Wako Pure Chemicals (Osaka, Japan). Morroniside was purchased from NPC BioTechnology Inc. (Daejeon, Korea). The purity of each component was determined to be above 98% by high performance liquid chromatography (HPLC) analysis. HPLC-grade reagents, methanol, acetonitrile, and water were obtained from J. T. Baker (Phillipsburg, NJ, USA). Acetic acid was procured from Junsei (Tokyo, Japan).

### 2.2. Plant Materials

Each of the six herbal medicines comprising YMJ was purchased from Omniherb (Yeongcheon, Korea) and HMAX (Chungbuk, Korea). The sources of these herbal medicines were confirmed taxonomically by Professor Je Hyun Lee, Dongguk University, Gyeongju, Korea. A voucher specimen (2008–KE07–1~KE07–6) has been deposited at the Herbal Medicine Formulation Research Group, Korea Institute of Oriental Medicine.

### 2.3. Preparation of YMJ Water Extract

A YMJ decoction consisting of six herbs (*Rehmannia glutinosa*,* Cornus officinalis*,* Dioscorea batatas*,* Paeonia suffruticosa*,* Poria cocos*, and* Alisma orientale*) was mixed (Table 1; 20.6 kg; 25.0 g × 825) and extracted in a 10-fold mass of water at 100°C for 2 h under pressure (1 kgf/cm^2^) using an electric extractor (COSMOS-660; Kyungseo Machine Co., Incheon, Korea), and the water extract was then filtered through a standard sieve (number 270, 53 *μ*m; Chung Gye Sang Gong Sa, Seoul, Korea) and the solution was evaporated to dryness and freeze-dried to give a powder. The yield of YMJ water extract was 27.0% (5.6 kg).

### 2.4. Preparation of Standard and Sample Solutions

Standard stock solutions of 5-HMF, gallic acid, paeoniflorin, paeonol, loganin, and morroniside were dissolved in methanol at 1.0 mg/mL and stored below 4°C. Working standard solutions were prepared by serial dilution of stock solutions with methanol. For HPLC analysis, 200 mg of lyophilized YMJ was dissolved in 20 mL of distilled water and passed through a 0.2 *μ*m syringe filter (Woongki Science, Seoul, Korea) before injection into the HPLC system.

### 2.5. HPLC Analysis of YMJ Sample

The chromatographic analysis for simultaneous determination used a Shimadzu Prominence LC-20A system (Shimadzu Co., Kyoto, Japan), consisting of a solvent delivery unit, an online degasser, a column oven, an autosampler, and a photodiode array (PDA) detector. The data processor employed Shimadzu LCsolution software (version 1.24). All analytes were separated on a Phenomenex Gemini C_18_ column (250 × 4.6 mm, 5 *μ*m, Torrance, CA) maintained at 40°C. The mobile phases consisted of 1.0% (v/v) aqueous acetic acid (A) and 1.0% (v/v) acetic acid in acetonitrile (B). The gradient flow was as follows: 5–40% B for 0–30 min; 40–100% B for 30–40 min; 100% B for 40–45 min; and 100–5% B for 50 min. The flow-rate was 1.0 mL/min and the injection volume was 10 *μ*L. The wavelength range of PDA detection was 190–400 nm and the detected wavelengths were monitored at 230 nm (morroniside, loganin, and paeoniflorin) or 280 nm (gallic acid, 5-HMF, and paeonol). All calibration curves were obtained by assessment of peak areas from standard solutions in the following concentration ranges: 5-HMF and morroniside, 3.13–200.00 *μ*g/mL; gallic acid and loganin, 0.78–50 *μ*g/mL; and paeoniflorin and paeonol, 0.16–10.00 *μ*g/mL.

### 2.6. Animals

The animal studies were conducted according to the guidance of the Institutional Animal Care and Use Committee in the Korea Institute of Toxicology (KRICT) (accredited by AAALAC International, 1998) under the current Good Laboratory Practice regulations for nonclinical laboratory studies and approved by the Korea Institute of Oriental Medicine Institutional Animal Care and Use Committee (Daejeon, Korea). Specific pathogen-free Crl:CD Sprague-Dawley (SD) rats (*n* = 24/gender) were obtained from Orient Bio Co. (Seoul, Korea) and used after 2 weeks of quarantine and acclimatization. The animals were housed in a room maintained at 22 ± 3°C under a relative humidity of 50 ± 20% with artificial lighting from 08:00 to 20:00 and 12–15 air changes per hour. The animals were kept in stainless-steel wire-mesh cages and allowed sterilized tap water and commercial rodent chow (PMI Nutrition International, Richmond, VA, USA) ad libitum.

### 2.7. Group Assignment and Treatment

Healthy male and female rats were assigned to four groups (*n* = 5/group) using Path/Tox System  4.2.2 (Xybion Medical Systems Corp., Cedar Knolls, NJ). YMJ extract was dissolved in distilled water for injection (Choong-Wae Pharmaceutical, Ltd., Korea) and administered by oral gavage at doses of 0, 500, 1000, and 2000 mg/kg/day for 4 weeks. Distilled water was given to the animals as the vehicle control. The daily dose (10 mL/kg body weight) of YMJ was calculated based on the most recently recorded body weights of individual animals.

### 2.8. General Observations

Clinical signs and any mortality were recorded twice a day (before and after treatment) throughout the study period. All clinical signs were recorded individually for type, observation day/time, and duration using Path/Tox System  4.2.2 (Xybion Medical Systems Corp.). The body weight of each rat was measured at the initiation of treatment and once a week during the study period. Food consumption was measured at the start of treatment and weekly throughout. Daily food consumption was determined by measuring the weights of chow supplied and remaining each day. External eye examination was carried out during the last week of treatment with an indirect binocular ophthalmoscope (IO-H, Neitz Instrument Co., Tokyo, Japan), and the appearance of the conjunctiva, sclera, cornea, lens, and iris of each eye was recorded.

### 2.9. Urinalysis, Hematology, and Serum Biochemistry

During the last week of treatment, urinalysis was conducted on samples collected overnight using a Multistix 10 SG (Bayer, Pittsburg, PA) and Clinitek-500 urine chemistry analyzer (Siemens Healthcare Diagnostics, Erlangen, Germany). Analysis included the volume, specific gravity (SG), pH, protein, ketone body (KET) level, any occult blood (BLO), glucose, and bilirubin (BIL), nitrite (NIT), and urobilinogen (URO) levels, and sediment.

Animals were fasted overnight prior to blood collection or necropsy. Blood was drawn from the posterior vena cava vein with the animals under isoflurane anesthesia. Samples were collected in CBC bottles containing EDTA-2K (Sewon Medical Co., Korea) and were analyzed to determine the red blood cell (RBC) count, the white blood cell (WBC) count, differential WBC count, hemoglobin concentration (HGC), hematocrit (HCT), mean corpuscular volume (MCV), mean corpuscular hemoglobin (MCH), mean corpuscular hemoglobin concentration (MCHC), and platelet (PLT) and reticulocyte (RET) counts using an ADVIA120 Hematology System (Bayer). Prothrombin time (PT) and activated partial thromboplastin time (APTT) were determined in blood samples treated with 3.2% sodium citrate using a coagulometer (ACL 300 plus, Instrumentation Laboratory SpA, Milan, Italy).

For serum biochemistry, blood samples were centrifuged at 3000 rpm for 10 min and analyzed with an autoanalyzer (Toshiba 200FR NEO, Toshiba Co., Japan). The analysis included the concentrations of alanine aminotransferase (ALT), aspartate aminotransferase (AST), alkaline phosphatase (ALP), gamma glutamyl transpeptidase (GGT), blood urea nitrogen (BUN), creatinine (CREA), creatine kinase (CK), glucose (GLU), total cholesterol (TCHO), albumin (ALB), albumin/globulin ratio (A/G), total protein (TP), triglyceride (TG), total bilirubin (TBIL), phospholipids (PL), sodium (Na), potassium (K), calcium (Ca), chloride (Cl), and inorganic phosphorus (IP).

### 2.10. Necropsy

All surviving animals were anesthetized with isofluorane and euthanized by aortic exsanguination prior to necropsy. Complete gross postmortem examinations were performed on all animals. Absolute organ weights were measured and relative organ weights (organ-to-body weight ratios) were calculated for the following organs: brain, pituitary gland, adrenal gland, liver, spleen, kidneys, heart, thymus, lung, salivary gland, thyroids, testes, ovaries, epididymides, seminal vesicle, prostate, and uterus.

### 2.11. Cytotoxicity Assay

Twenty-three various cell lines were obtained from the American Type Culture Collection (ATCC, Rockville, MD, USA) or the Korean Cell Line Bank (Seoul, Korea). Cytotoxic effects of YMJ against the cell lines were measured using a 3-(4,5-dimethylthiazol-2-yl)-2,5-diphenyl tetrazolium bromide (MTT) assay as described [10]. Cell viability was calculated as the percentage of viable cells in the drug-treated group versus untreated controls based on optical density (OD) from the following equation: Cell viability = [OD (YMJ) − OD (Blank)]/[OD (Control) − OD (Blank) ] × 100.

### 2.12. Statistical Analyses

Data collected during the study were examined for homogeneity of variance using Bartlett's test. When Bartlett's test indicated no significant deviation from homogeneity, a one-way analysis of variance (ANOVA) was performed at *α* = 0.05. When significance was noted, a multiple comparison test (Dunnett's test) was conducted to determine which pairs of groups were significantly different. Where significant deviations from homogeneity of variance were observed, a nonparametric comparison test (Kruskal-Wallis test) was conducted. When a significant difference was observed in the Kruskal-Wallis test, Dunn's rank sum test was conducted to determine the specific pairs. Statistical analyses were performed using the Path/Tox System (ver. 4.2.2): Xybion Medical Systems Corp.). The level of significance was taken as *P* < 0.05 or *P* < 0.01.

## 3. Results

### 3.1. Characterization of the YMJ Extract

**Figure 1:**
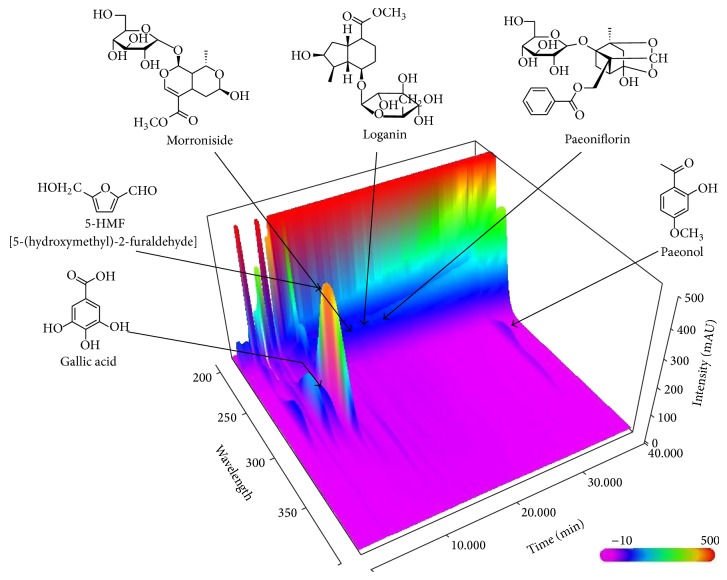
Three-dimensional chromatogram of YMJ by HPLC-PDA. Column: Gemini C_18_ (250 × 4.6 mm, 5 *μ*m); mobile phase: 1.0% (v/v) acetic acid in water (A) and 1.0% (v/v) acetic acid in acetonitrile (B) (5–40% B for 0–30 min, 40–100% B for 30–40 min, 100% B for 40–45 min, and 100–5% B for 50 min); flow rate: 1.0 mL/min; retention time: 5.95 min (gallic acid), 8.09 min (5-HMF), 13.14 min (morroniside), 16.15 min (loganin), 18.30 min (paeoniflorin), and 36.00 min (paeonol).

The linearity of the method was evaluated from the correlation coefficient (*r* ^2^) of the calibration curves of each compound. We found that the six compounds showed good linearity with *r* ^2^ ≥ 0.9997 in seven different concentration ranges. The limit of detection (LOD) and limit of quantification (LOQ) ranges of the six tested compounds were 0.01–0.08 *μ*g/mL and 0.02–0.27 *μ*g/mL, respectively (Table 2). Using optimized chromatography conditions, three-dimensional chromatograms were obtained using the HPLC-PDA detector (Figure 1). The retention times of the components were 5.95 min (gallic acid), 8.09 min (5-HMF), 13.14 min (morroniside), 16.15 min (loganin), 18.30 min (paeoniflorin), and 36.00 min (paeonol). The concentration range of the six biomarker components was 1.15–4.32 mg/g (Table 3).

### 3.2. Clinical Signs and Mortality

No treatment-related clinical sign was observed in any of the rats (Table 4). In addition, no animal death was recorded during treatment for 4 weeks (Table 5).

### 3.3. Body Weight Changes and Food Intake

**Figure 2:**
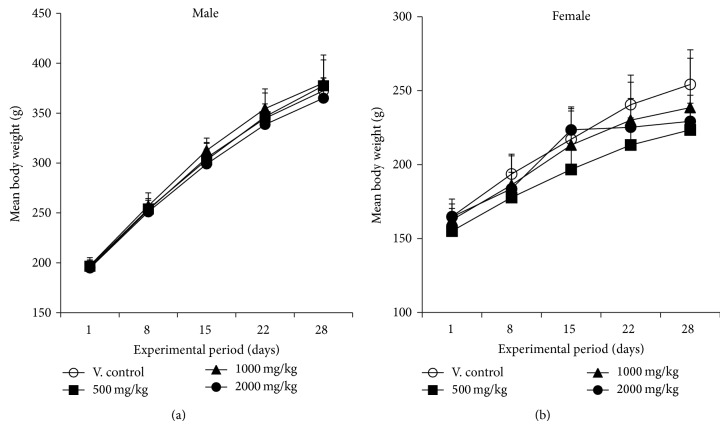
Mean body weight changes of male (a) and female (b) rats treated with YMJ. Healthy male and female rats were assigned to four groups (*n* = 5/group) using Path/Tox System  4.2.2. YMJ extract was dissolved in distilled water for injection and administered by oral gavage at doses of 0 (○), 500 (■), 1,000 (▲), and 2,000 (●) mg/kg/day for 4 weeks. Distilled water was given to the animals as the vehicle control. Values are presented as the mean ± SD.

**Figure 3:**
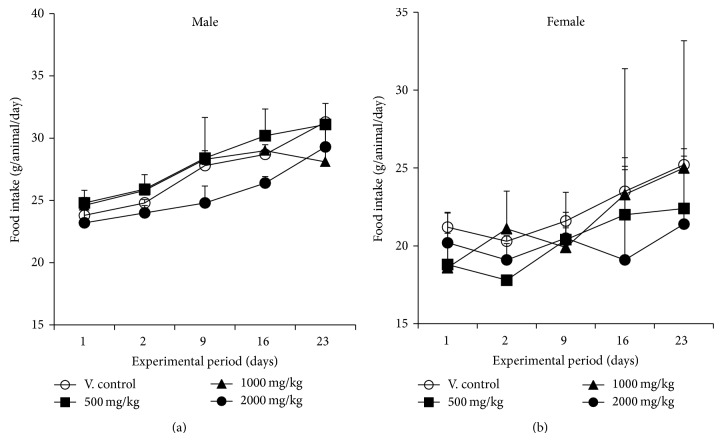
Food intake in male (a) and female (b) rats treated with YMJ. Healthy male and female rats were assigned to four groups (*n* = 5/group) using Path/Tox System  4.2.2. YMJ extract was dissolved in distilled water for injection and administered by oral gavage at doses of 0 (○), 500 (■), 1,000 (▲), and 2,000 (●) mg/kg/day for 4 weeks. Distilled water was given to the animals as the vehicle control. Values are the mean ± SD. ^*^ *P* < 0.05 versus vehicle control group (V. control).

In both male and female rats treated with YMJ, there were no significant differences in body weight between vehicle control and YJM-treated groups (Figure 2). No significant differences in food consumption were observed between vehicle control and treatment group for either gender (Figure 3).

### 3.4. Necropsy

Absolute organ weights were measured and the relative organ weight (organ weight/fasted body weight) was calculated for 15 different organs (Table 6). In male rats, a decrease in the absolute weight of the epididymides was noted at 1000 and 2000 mg/kg/day (one-way ANOVA; *P* < 0.05). In contrast, the absolute weights of all tested organs had no significant differences between vehicle control and YMJ-treated female rats (Table 6). In addition, no treatment-related gross finding was observed in the necropsy performed at euthanasia for both genders (data not shown).

### 3.5. Hematology, Serum Biochemistry, and Urinalysis

No significant differences in hematology test outcomes were found between vehicle control and YMJ-treated male rats (Table 7). In contrast, PT was significantly reduced in female rats at 1000 and 2000 mg/kg/day (one-way ANOVA; *P* < 0.01 and *P* < 0.05, resp.). As shown in Table 8, serum biochemical analysis showed that the Na concentration was increased in YMJ-treated male rats at 1000 mg/kg/day (Kruskal-Wallis test; *P* < 0.05) whereas the creatine level was decreased in YMJ-fed female rats at 500 and 1000 mg/kg/day (Kruskal-Wallis test; *P* < 0.05 and *P* < 0.01, resp.). There were no significant differences in urinalysis values between the vehicle-only control and treatment groups (data not shown).

### 3.6. Cytotoxicity

Cytotoxicity of YMJ was evaluated against 23 different cell lines. Cells were treated with various concentrations (0, 10, 20, 50, 100, or 200 *μ*g/mL) of YMJ extract for 24 h. As shown in Table 9, the cell viability was not significantly altered in any of the tested cell lines including SH-SY5Y, SK-N-SH (neuroblastomas), U-373 MG, U-87 MG (glioblastomas), HepG2, Hep3B (hepatocarcinomas), Clone M-3, B16F10 (melanomas), HEK-293, NRK52 (kidney cells), 3T3-L1, NIH3T3 (fibroblasts), HIT-T15 (pancreatic cells), HL-60, RBL-1 (leukemias), HT-29 (colon cancer), MCF-7 (breast cancer), HaCaT (keratinocytes), PC12 (phechromocytomas), LNCaP (prostate cancer), BEAS-2B (bronchial epithelial cells), and AGS (gastric adenocarcinoma) up to 200 *μ*g/mL in all tested cell lines.

## 4. Discussion

YMJ is a well-known herbal formula that has long been used in CAM for the treatment of renal diseases and diabetes mellitus. Several studies have provided scientific evidence on the biological activities of YMJ against renal diseases, diabetes, and bone diseases. Thus, treatment with YMJ ameliorated renal defects in rats with ischemia/reperfusion-induced acute renal failure (ARF) [11]. YMJ markedly restored renal functional parameters including creatine clearance, urinary sodium excretion, urinary osmolality, and solute-free reabsorption with lowering the expression of renal aquaporin 2 (AQP2) and heme oxygenase 1 (HO-1). Additionally, YMJ pills induced apoptosis by regulating the expression of the bcl-2/bax genes at the transcriptional level in the pancreas of Otsuka Long-Evans Tokushima fatty (OLETF) rats with type 2 diabetes [10]. Shim et al. reported the therapeutic potential of YMJ in treating bone diseases by suppressing receptor activator for nuclear factor-*κ*B ligand- (RANK-) induced osteoclast differentiation [3]. Moreover, Wang et al. reported the combinatorial effect of YMJ and antihypertensive drugs on the improvement of blood pressure and symptoms, suggesting a new integrative medicine therapy for patients with essential hypertension patients [12].

Given the efficacy of YMJ, information on its safety as a medicine should be evaluated carefully because YMJ is a mixture of six different herbs as well as several different phytochemicals. Previously, we reported on the acute and subchronic toxicity as well as genotoxicity of YMJ in an animal model. We found that YMJ had no acute and subchronic toxicity or genotoxicity and its no-observed-adverse-effect-level (NOAEL) is 2000 mg/kg/day [13, 14]. However, there has been no report on the subacute toxicity of YMJ. Therefore, here we evaluated the toxicity of YMJ over 4 weeks to test its subacute toxicity in male or female Crl:CD (SD) rats.

We observed that YMJ did not significantly change clinical signs, body weight, or food intake in either male or female rats. The Society of Toxicologic Pathology (STP) recommends the evaluation of organ weights in general toxicology studies [15]. However, YMJ treatment decreased the absolute weights of both epididymides at 1000 and 2000 mg/kg/day in male rats. Epididymal weight changes are a sensitive indicator of decreased sperm production and can reflect edema or inflammation. Thus, epididymal weight should be measured routinely for multidose rat toxicology tests using GLP [16]. However, the changes in epididymal weights caused by YMJ treatment were not severe and not dose-dependent and were considered to be in the normal physiological range [17].

In terms of hematological data, YMJ administration significantly decreased the PT at 1000 and 2000 mg/kg/day after female rats. A prolonged PT is an early indicator of renal damage in poisoning with metabolic toxins [18]. The reference range for PT is usually less than 20 sec depending on the assay protocol [19]. In our study, the PT values of YMJ-treated rats were 13.2 and 13.4 sec at 1000 and 2000 mg/kg, respectively, namely, in the normal range. Serum biochemical values are also important parameters in toxicology studies [20]. We found that YMJ treatment increased the Na concentration at 1000 mg/kg/day in male rats and decreased the creatine levels at 500 and 1000 mg/kg/day in female rats. Serum Na concentration reflects the pump that maintains the constancy of its extracellular concentration and creatine content reflects renal functional capacity [21]. Changes in Na and creatine contents of the serum in our study did not exceed the normal range (Na: 143–156 mEq/L, creatine: 0.2–0.8 mg/dL) [22].

Thus, 4-week repeated oral administration of YMJ caused no subacute toxic effects in rats. Consistent with the results of other toxicity studies [23, 24], the NOAEL was estimated to be 2000 mg/kg/day. In the pharmacokinetic aspect, the metabolism rate in small animals including mouse and rat is faster than in humans [25]. As we previously reported [13], the equivalent dose of YMJ in rats converted from its single dose in humans was 1,329 mg according to van Miert [26], which is a value less than the NOAEL (2000 mg/kg in rats). The single administration dose of YMJ in human is 46.875 g dried herb [1], which is equivalent to 12.656 g of the extract (yield = 27.0% of the raw material).

Furthermore, we tested the cytotoxicity of YMJ against 23 different cell lines (Table 9). The cells were treated with YMJ extract at a concentration range of 1–200 *μ*g/mL for 24 h. YMJ had no effect on the viability of several cancer cell lines including neuroblastomas (SH-SY5Y and SK-N-SH), glioblastomas (U-373 MG and U-87 MG), hepatocarcinomas (HepG2 and Hep3B), melanomas (Clone M-3 and B16F10), leukemias (HL-60 and RBL-1), colon cancer (HT-29), breast cancer (MCF-7), phechromocytomas (PC12), prostate cancer (LNCaP), or gastric adenocarcinomas (AGS). Nor did YMJ treatment induce any toxicity in kidney cells (HEK-293 and NRK52), fibroblasts (3T3-L1 and NIH3T3), pancreatic cells (HIT-T15), keratinocytes (HaCaT), and bronchial epithelial cells (BEAS-2B). These results could be used when evaluating the biological activity of YMJ using* in vitro* assays.

In conclusion, our findings provide information on the safety of YMJ* in vitro* and* in vivo*. Using a model of SD rats fed with YMJ for 4 weeks, we demonstrated that YMJ did not have severe or specific adverse effects in either male or female rats at doses of up to 2000 mg/kg/day. Our* in vitro *cytotoxicity study also showed that YMJ had no significant effects on the viability of 23 different cell lines. Our data and other information on YMJ safety could be valuable to establish preclinical and clinical studies for YMJ or its related herbal formulas. Furthermore, many studies have reported that the six herbal components (*Rehmannia glutinosa, Cornus officinalis, Dioscorea batatas, Paeonia suffruticosa, Poria cocos, *and* Alisma orientale*) have biological potential as preventive or therapeutic agents. Therefore, we consider that the efficacy of YMJ probably results from synergistic interactions between the herbal components; it will therefore be necessary to define the safety of each component. In addition, studies should be conducted in suitable nonrodent model before extrapolating the results to human medicine.

## Figures and Tables

**Table 1:** Composition of YMJ.

Scientific name	Amount (g)	Supplier	Source
*Rehmannia glutinosa *	8.0	Omniherb	Kunwi, Korea
*Cornus officinalis *	4.0	Omniherb	Gurye, Korea
*Dioscorea batatas *	4.0	Omniherb	Kunwi, Korea
*Paeonia suffruticosa *	3.0	HMAX	P. R. China
*Poria cocos *	3.0	Omniherb	Yeongcheon, Korea
*Alisma orientale *	3.0	Omniherb	Imsil, Korea

Total	25.0		

**Table 2:** Regression equations, linearity, LOD, and LOQ for six standard compounds of YMJ.

Analyte	Linear range (*μ*g/mL)	Regression equation^a^	Correlation coefficient (*r* ^2^)	LOD^b^ (*μ*g/mL)	LOQ^c^ (*μ*g/mL)
Gallic acid	0.78–50.00	*y* = 26606.11*x* − 8860.81	0.9998	0.02	0.06
5-HMF	3.13–200.00	*y* = 82889.12*x* − 2299.96	0.9999	0.01	0.02
Morroniside	3.13–200.00	*y* = 18597.16*x* − 9630.17	1.0000	0.04	0.14
Loganin	0.78–50.00	*y* = 16410.59*x* − 1454.52	1.0000	0.05	0.17
Paeoniflorin	0.16–10.00	*y* = 10461.75*x* − 4859.01	0.9997	0.08	0.27
Paeonol	0.16–10.00	*y* = 48319.49*x* + 86.82	0.9999	0.05	0.16

^a^ *y*: peak area (mAU) of compounds; *x*: concentration (*μ*g/mL) of compounds.

^b^LOD: 3 × signal-to-noise ratio.

^c^LOQ: 10 × signal-to-noise ratio.

**Table 3:** Contents of six standard components in YMJ measured by HPLC (*n* = 3).

Component	Mean (mg/g)	SD	RSD (%)	Source
Gallic acid	1.70	0.01	0.31	*Paeonia suffruticosa *
5-HMF	4.32	0.01	0.31	*Rehmannia glutinosa *
Morroniside	2.69	0.02	0.59	*Cornus officinalis *
Loganin	1.63	0.02	1.14	*Cornus officinalis *
Paeoniflorin	1.15	0.03	2.22	*Paeonia suffruticosa *
Paeonol	1.32	0.00	0.18	*Paeonia suffruticosa *

**Table 4:** Clinical signs in rats treated orally with YMJ.

Group	Salivation	Scabbing
Male rats		
Vehicle control	0/5	0/5
500 mg/kg	0/5	0/5
1000 mg/kg	0/5	0/5
2000 mg/kg	0/5	0/5
Female rats		
Vehicle control	0/5	0/5
500 mg/kg	0/5	0/5
1000 mg/kg	0/5	0/5
2000 mg/kg	0/5	0/5

**Table 5:** Mortality in rats treated orally with YMJ.

Dose (mg/kg)	Dosing phase					Final mortality
	1 day	≤7 days	≤14 days	≤21 days	≤29 days	
0	0	0	0	0	0	0/5
500	0	0	0	0	0	0/5
1000	0	0	0	0	0	0/5
2000	0	0	0	0	0	0/5

**Table 6:** Absolute organ weights in rats treated with YMJ for 4 weeks.

Dose (mg/kg/day)	0	500	1000	2000
Male rats				
Brain	1.919 ± 0.0808	1.880 ± 0.1136	1.949 ± 0.0403	1.945 ± 0.0833
Pituitary gland	0.010 ± 0.0026	0.010 ± 0.0023	0.010 ± 0.0022	0.009 ± 0.0028
Liver	10.102 ± 0.5764	10.403 ± 0.7475	10.483 ± 0.1410	10.234 ± 1.5110
Spleen	0.607 ± 0.0700	0.640 ± 0.0979	0.725 ± 0.0848	0.643 ± 0.1482
Heart	1.180 ± 0.0643	1.172 ± 0.1247	1.173 ± 0.0273	1.110 ± 0.1174
Thymus	0.558 ± 0.1072	0.600 ± 0.0548	0.492 ± 0.0949	0.550 ± 0.1330
Salivary glands	0.625 ± 0.0561	0.581 ± 0.0779	0.593 ± 0.0475	0.547 ± 0.0688
Seminal vesicle	1.031 ± 0.1515	1.089 ± 0.1220	0.944 ± 0.1084	0.994 ± 0.1193
Prostate	0.468 ± 0.1143	0.405 ± 0.1367	0.405 ± 0.0543	0.402 ± 0.0394
Kidneys	2.501 ± 0.1742	2.506 ± 0.1711	2.535 ± 0.0996	2.498 ± 0.2781
Adrenal glands	0.052 ± 0.0073	0.046 ± 0.0044	0.049 ± 0.0073	0.047 ± 0.0060
Testes	3.237 ± 0.2174	2.971 ± 0.2266	3.177 ± 0.1425	3.023 ± 0.1289
Epididymides	0.911 ± 0.0681	0.893 ± 0.0425	0.831 ± 0.0345^*^	0.815 ± 0.0289^*^
Lung	1.317 ± 0.0284	1.313 ± 0.1563	1.359 ± 0.0700	1.278 ± 0.1018
Thyroid/parathyroid	0.016 ± 0.0053	0.015 ± 0.0046	0.014 ± 0.0025	0.015 ± 0.0047
Female rats				
Brain	1.780 ± 0.0734	1.740 ± 0.0536	1.825 ± 0.0755	1.758 ± 0.0998
Pituitary gland	0.010 ± 0.0034	0.010 ± 0.0025	0.010 ± 0.0023	0.011 ± 0.0017
Liver	7.387 ± 0.7843	6.397 ± 0.5466	7.076 ± 1.3626	6.948 ± 0.5587
Spleen	0.504 ± 0.0368	0.451 ± 0.0938	0.511 ± 0.1144	0.543 ± 0.0822
Heart	0.889 ± 0.1013	0.838 ± 0.0615	0.864 ± 0.1392	0.825 ± 0.0920
Thymus	0.455 ± 0.0396	0.433 ± 0.0476	0.447 ± 0.1072	0.460 ± 0.0314
Salivary glands	0.426 ± 0.0514	0.390 ± 0.0262	0.418 ± 0.0558	0.376 ± 0.0244
Kidneys	1.814 ± 0.1545	1.731 ± 0.1631	1.880 ± 0.2300	1.684 ± 0.1485
Adrenal glands	0.066 ± 0.0088	0.059 ± 0.0090	0.067 ± 0.0108	0.068 ± 0.0123
Ovaries	0.087 ± 0.0092	0.079 ± 0.0223	0.092 ± 0.0311	0.088 ± 0.0194
Lung	1.094 ± 0.0422	1.042 ± 0.0847	1.034 ± 0.1157	1.090 ± 0.1078
Thyroid/parathyroid	0.012 ± 0.0048	0.011 ± 0.0025	0.015 ± 0.0023	0.014 ± 0.0037
Uterus/cervix	0.614 ± 0.3916	0.458 ± 0.1359	0.617 ± 0.2779	0.402 ± 0.0820

Values are presented as the mean ± SD.

^*^ *P* < 0.05 compared with the vehicle control.

**Table 7:** Hematological values of animals treated with YMJ for 4 weeks.

Dose (mg/kg/day)	0	500	1000	2000
Male rats				
WBC (10^3^/*μ*L)	13.17 ± 3.558	11.65 ± 3.265	14.15 ± 3.582	10.30 ± 3.453
Reticulocytes (%)	2.7 ± 0.33	2.4 ± 0.21	2.5 ± 0.31	2.6 ± 0.56
Neutrophils (%)	15.72 ± 4.759	13.54 ± 3.412	11.46 ± 3.400	13.06 ± 2.859
Lymphocytes (%)	78.9 ± 5.44	81.1 ± 2.97	83.0 ± 4.06	81.6 ± 2.72
Eosinophils (%)	0.9 ± 0.40	0.8 ± 0.18	0.8 ± 0.24	0.9 ± 0.57
Monocytes (%)	3.1 ± 0.54	3.2 ± 0.85	3.2 ± 0.97	3.1 ± 0.61
Basophils (%)	0.3 ± 0.04	0.3 ± 0.07	0.2 ± 0.00	0.3 ± 0.13
Large unstained cells (%)	1.2 ± 0.33	1.0 ± 0.23	1.3 ± 0.52	1.1 ± 0.31
RBC (10^6^/*μ*L)	7.92 ± 0.182	8.02 ± 0.458	7.75 ± 0.149	7.87 ± 0.321
Hemoglobin (g/dL)	15.5 ± 0.37	15.5 ± 0.58	15.4 ± 0.25	15.6 ± 0.55
Hematocrit (%)	46.3 ± 1.09	46.3 ± 1.70	45.8 ± 0.60	46.5 ± 1.49
MCV (fL)	58.4 ± 0.86	57.8 ± 1.65	59.2 ± 1.50	59.2 ± 1.71
MCH (pg)	19.5 ± 0.27	19.3 ± 0.74	20.0 ± 0.57	19.9 ± 0.30
MCHC (g/dL)	33.5 ± 0.62	33.4 ± 0.38	33.7 ± 0.40	33.6 ± 0.60
Platelet (10^3^/*μ*L)	1312 ± 107.8	1112 ± 89.7	1258 ± 168.9	1192 ± 132.3
PT (sec)	13.7 ± 0.13	13.7 ± 0.48	13.6 ± 0.19	13.9 ± 0.25
Female rats				
WBC (10^3^/*μ*L)	9.28 ± 1.902	8.15 ± 1.819	8.79 ± 1.528	8.57 ± 0.896
Reticulocytes (%)	2.2 ± 0.35	1.9 ± 0.49	2.6 ± 0.85	2.0 ± 0.47
Neutrophils (%)	15.82 ± 4.190	15.70 ± 6.911	12.10 ± 4.552	19.00 ± 10.368
Lymphocytes (%)	78.7 ± 5.08	78.9 ± 6.51	82.7 ± 3.22	75.8 ± 10.93
Eosinophils (%)	0.9 ± 0.19	1.2 ± 0.24	1.2 ± 0.64	1.2 ± 0.15
Monocytes (%)	3.1 ± 1.17	3.0 ± 1.68	2.4 ± 0.92	2.8 ± 0.93
Basophils (%)	0.3 ± 0.09	0.2 ± 0.12	0.3 ± 0.04	0.2 ± 0.05
Large unstained cells (%)	1.2 ± 0.15	1.0 ± 0.25	1.3 ± 0.48	0.9 ± 0.32
RBC (10^6^/*μ*L)	7.63 ± 0.187	7.76 ± 0.313	7.72 ± 0.287	7.76 ± 0.224
Hemoglobin (g/dL)	15.1 ± 0.29	15.2 ± 0.49	15.3 ± 0.23	15.3 ± 0.30
Hematocrit (%)	44.6 ± 0.38	44.8 ± 1.25	45.4 ± 0.82	44.6 ± 0.76
MCV (fL)	58.5 ± 1.41	57.8 ± 1.21	58.9 ± 2.99	57.5 ± 1.08
MCH (pg)	19.7 ± 0.49	19.7 ± 0.58	19.8 ± 0.83	19.8 ± 0.47
MCHC (g/dL)	33.7 ± 0.40	34.0 ± 0.61	33.7 ± 0.57	34.4 ± 0.24
Platelet (10^3^/*μ*L)	1157 ± 74.5	1329 ± 119.5	1158 ± 71.5	1223 ± 127.7
PT (sec)	14.0 ± 0.29	13.8 ± 0.41	13.2 ± 0.18^**^	13.4 ± 0.51^*^

MCV, mean corpuscular volume; MCH, mean corpuscular hemoglobin; MCHC, mean corpuscular hemoglobin concentration; PT, prothrombin time.

Values are presented as the mean ± SD.

^*^ *P* < 0.05 and ^**^ *P* < 0.01 compared with the vehicle control group.

**Table 8:** Serum biochemical values of animals treated with YMJ for 4 weeks.

Dose (mg/kg/day)	0	500	1000	2000
Male rats				
Glucose (mg/dL)	115.9 ± 13.28	102.8 ± 9.00	102.3 ± 18.22	117.3 ± 17.18
BUN (mg/dL)	10.9 ± 0.84	12.2 ± 0.66	11.7 ± 1.90	12.4 ± 1.35
Creatinine (mg/dL)	0.49 ± 0.057	0.48 ± 0.015	0.52 ± 0.055	0.51 ± 0.040
Total protein (g/dL)	6.25 ± 0.173	6.18 ± 0.308	6.41 ± 0.304	6.33 ± 0.045
Albumin (g/dL)	4.30 ± 0.084	4.28 ± 0.169	4.38 ± 0.105	4.36 ± 0.067
Albumin/globulin ratio	2.21 ± 0.094	2.26 ± 0.121	2.18 ± 0.204	2.23 ± 0.145
Total cholesterol (mg/dL)	61.4 ± 8.99	57.8 ± 9.28	60.6 ± 12.30	60.8 ± 10.85
Triglycerides (mg/dL)	44.7 ± 16.71	49.1 ± 18.69	44.5 ± 14.46	49.4 ± 22.12
Phospholipid (mg/dL)	98 ± 10.7	92 ± 9.9	95 ± 13.9	95 ± 19.4
AST (IU/L)	108.1 ± 8.07	116.7 ± 14.85	121.2 ± 20.65	114.2 ± 14.18
ALT (IU/L)	28.8 ± 4.09	31.5 ± 4.90	35.9 ± 12.72	27.2 ± 1.25
Total bilirubin (mg/dL)	0.096 ± 0.0113	0.105 ± 0.0092	0.112 ± 0.0116	0.098 ± 0.0067
ALP (IU/L)	449.6 ± 34.20	491.2 ± 84.72	476.0 ± 28.92	479.6 ± 94.04
Creatine kinase (IU/L)	527 ± 124.0	639 ± 111.1	656 ± 168.0	637 ± 217.8
Ca (mg/dL)	10.89 ± 0.203	10.55 ± 0.376	10.78 ± 0.425	10.77 ± 0.479
IP (mg/dL)	10.43 ± 0.907	9.86 ± 1.044	10.04 ± 1.174	9.72 ± 0.924
Na (mmol/L)	144 ± 0.9	145 ± 0.7	146 ± 1.5^*^	146 ± 0.7
K (mmol/L)	7.52 ± 0.800	6.51 ± 1.190	5.80 ± 1.195	6.49 ± 1.255
Cl (mmol/L)	103 ± 1.9	103 ± 1.1	103 ± 1.3	104 ± 2.1
GGT (IU/L)	1.03 ± 1.460	0.15 ± 0.143	0.15 ± 0.143	0.44 ± 0.615
Female rats				
Glucose (mg/dL)	110.6 ± 17.45	105.5 ± 15.58	118.9 ± 12.20	91.1 ± 7.68
BUN (mg/dL)	15.76 ± 2.25	13.6 ± 0.82	14.0 ± 2.02	14.4 ± 1.43
Creatinine (mg/dL)	0.60 ± 0.011	0.55 ± 0.007^*^	0.54 ± 0.011^**^	0.59 ± 0.029
Total protein (g/dL)	6.76 ± 0.451	6.65 ± 0.255	6.82 ± 0.224	6.76 ± 0.381
Albumin (g/dL)	4.58 ± 0.243	4.65 ± 0.136	4.65 ± 0.118	4.61 ± 0.205
Albumin/globulin ratio	2.10 ± 0.114	2.34 ± 0.120	2.15 ± 0.103	2.16 ± 0.168
Total cholesterol (mg/dL)	70.0 ± 9.72	70.0 ± 12.98	72.6 ± 13.87	75.8 ± 21.81
Triglycerides (mg/dL)	36.5 ± 6.90	34.3 ± 3.47	30.2 ± 7.55	31.6 ± 7.08
Phospholipid (mg/dL)	123 ± 20.2	124 ± 15.1	127 ± 21.5	129 ± 24.9
AST (IU/L)	109.3 ± 17.78	108.7 ± 20.14	101.1 ± 10.93	116.5 ± 24.99
ALT (IU/L)	27.1 ± 4.47	25.9 ± 2.50	24.4 ± 4.06	27.0 ± 3.28
Total bilirubin (mg/dL)	0.112 ± 0.0101	0.116 ± 0.0111	0.118 ± 0.0251	0.117 ± 0.0178
ALP (IU/L)	217.9 ± 20.72	244.9 ± 15.87	261.3 ± 61.05	290.1 ± 99.47
Creatine kinase (IU/L)	498 ± 172.4	518 ± 166.8	387 ± 93.7	520 ± 233.7
Ca (mg/dL)	10.82 ± 0.444	10.80 ± 0.344	11.14 ± 0.316	10.97 ± 0.418
IP (mg/dL)	8.30 ± 0.427	7.84 ± 0.926	8.49 ± 0.770	8.80 ± 0.329
Na (mmol/L)	143 ± 1.6	144 ± 1.3	145 ± 1.1	143 ± 0.8
K (mmol/L)	6.06 ± 0.394	6.23 ± 1.241	6.52 ± 0.350	6.86 ± 0.573
Cl (mmol/L)	104 ± 2.4	105 ± 1.3	104 ± 1.3	103 ± 1.1
GGT (IU/L)	0.92 ± 0.579	0.67 ± 0.524	0.99 ± 0.603	0.99 ± 0.374

ALP, alkaline phosphatase; AST, aspartate aminotransferase; ALT, alkaline phosphatase; BUN, blood urea nitrogen.

Values are presented as mean ± SD.

^*^ *P* < 0.05 and ^**^ *P* < 0.01 compared with the vehicle control group.

**Table 9:** Cytotoxicity of YMJ against various cell lines.

Cell line	Origin	Concentration (*μ*g/mL)					
		0	10	20	50	100	200
SH-SY5Y	Human, neuroblastoma	100 ± 1.84	97 ± 1.69	98 ± 0.92	96 ± 1.17	100 ± 2.40	101 ± 0.60
Clone M-3	Mouse, melanoma	100 ± 9.99	95 ± 5.17	95 ± 3.66	94 ± 4.67	94 ± 2.34	94 ± 1.40
SK-N-SH	Human, neuroblastoma (brain)	100 ± 4.14	101 ± 0.78	93 ± 4.01	102 ± 1.35	98 ± 1.70	98 ± 4.53
U-373 MG	Human, glioblastoma (brain)	100 ± 8.15	96 ± 8.06	97 ± 4.48	96 ± 8.70	95 ± 5.41	94 ± 6.69
U-87 MG	Human, glioblastoma (brain)	100 ± 3.03	97 ± 5.65	95 ± 3.67	95 ± 4.6	99 ± 5.39	97 ± 4.74
HEK-293	Human, kidney	100 ± 6.96	124 ± 3.74	126 ± 3.55	118 ± 2.11	117 ± 3.96	118 ± 2.37
HepG2	Human, hepatoblastoma	100 ± 3.40	95 ± 4.11	94 ± 4.42	92 ± 4.47	91 ± 4.44	90 ± 2.09
Hep3B	Human, hepatocarcinoma	100 ± 2.73	101 ± 1.43	100 ± 2.79	99 ± 3.13	97 ± 1.61	92 ± 0.86
RAW264.7	Mouse, macrophage	100 ± 2.15	107 ± 1.90	110 ± 2.02	109 ± 4.06	108 ± 6.42	108 ± 3.17
3T3-L1	Mouse, fibroblast (preadipocyte)	100 ± 1.68	101 ± 0.47	97 ± 1.58	98 ± 2.32	101 ± 3.11	92 ± 3.91
HIT-T15	Hamster, pancreas	100 ± 0.60	101 ± 2.10	100 ± 3.40	99 ± 2.50	98 ± 3.10	100 ± 2.30
HL-60	Human, leukemia, lymphoblast	100 ± 2.56	99 ± 3.30	100 ± 2.43	98 ± 1.82	99 ± 2.73	99 ± 2.61
HT-29	Human, colon cancer cell	100 ± 4.28	107 ± 7.36	104 ± 6.67	107 ± 14.16	105 ± 13.91	97 ± 4.00
NIH3T3	Mouse, fibroblast	100 ± 7.56	102 ± 9.10	101 ± 2.39	101 ± 1.93	101 ± 3.94	101 ± 8.42
B16F10	Mouse, melanoma	100 ± 6.86	101 ± 8.66	93 ± 6.32	102 ± 4.23	98 ± 4.84	98 ± 6.26
MCF-7	Human, breast cancer	100 ± 1.66	103 ± 2.45	105 ± 1.95	113 ± 3.67	114 ± 2.12	114 ± 1.47
HaCaT	Human, keratinocyte	100 ± 2.69	96 ± 2.08	90 ± 4.12	91 ± 3.59	89 ± 2.87	89 ± 2.90
PC12	Rat, adrenal medulla, pheochromocytoma	100 ± 2.92	109 ± 2.01	105 ± 0.42	105 ± 2.05	103 ± 1.19	106 ± 2.71
AGS	Human, gastric adenocarcinoma	100 ± 0.74	101 ± 0.92	94 ± 0.51	100 ± 0.80	95 ± 1.76	95 ± 0.79
BEAS-2B	Human, bronchial epithelial, normal cell	100 ± 5.09	102 ± 2.64	107 ± 4.50	93 ± 3.78	96 ± 1.54	88 ± 2.27
LNCaP	Human, prostate cancer	100 ± 8.39	106 ± 6.33	106 ± 6.92	104 ± 10.50	103 ± 10.95	105 ± 8.63
RBL-1	Rat, basophilic leukemia	100 ± 2.55	98 ± 2.89	94 ± 0.75	94 ± 1.39	94 ± 1.71	94 ± 0.88
NRK52	Rat, kidney	100 ± 1.96	122 ± 2.27	121 ± 2.96	121 ± 0.95	124 ± 1.55	123 ± 2.34
